# Variation in carbon and nitrogen concentrations among peatland categories at the global scale

**DOI:** 10.1371/journal.pone.0275149

**Published:** 2022-11-23

**Authors:** Shaun Watmough, Spencer Gilbert-Parkes, Nathan Basiliko, Louis J. Lamit, Erik A. Lilleskov, Roxanne Andersen, Jhon del Aguila-Pasquel, Rebekka E. Artz, Brian W. Benscoter, Werner Borken, Luca Bragazza, Stefani M. Brandt, Suzanna L. Bräuer, Michael A. Carson, Xin Chen, Rodney A. Chimner, Bev R. Clarkson, Alexander R. Cobb, Andrea S. Enriquez, Jenny Farmer, Samantha P. Grover, Charles F. Harvey, Lorna I. Harris, Christina Hazard, Alison M. Hoyt, John Hribljan, Jyrki Jauhiainen, Sari Juutinen, Evan S. Kane, Klaus-Holger Knorr, Randy Kolka, Mari Könönen, Anna M. Laine, Tuula Larmola, Patrick A. Levasseur, Carmody K. McCalley, Jim McLaughlin, Tim R. Moore, Nadia Mykytczuk, Anna E. Normand, Virginia Rich, Bryce Robinson, Danielle L. Rupp, Jasmine Rutherford, Christopher W. Schadt, Dave S. Smith, Graeme Spiers, Leho Tedersoo, Pham Q. Thu, Carl C. Trettin, Eeva-Stiina Tuittila, Merritt Turetsky, Zuzana Urbanová, Ruth K. Varner, Mark P. Waldrop, Meng Wang, Zheng Wang, Matt Warren, Magdalena M. Wiedermann, Shanay T. Williams, Joseph B. Yavitt, Zhi-Guo Yu, Geoff Zahn

**Affiliations:** 1 Trent University, School of the Environment, Peterborough, Ontario, Canada; 2 Department of Biology and the Vale Living with Lakes Centre, Laurentian University, Sudbury, Ontario, Canada; 3 Department of Biology, Syracuse University, Syracuse, NY, United States of America; 4 USDA Forest Service, Northern Research Station, Houghton, MI, United States of America; 5 Environmental Research Institute, University of the Highlands and Islands, Castle St., United Kingdom; 6 Instituto de Investigaciones de la Amazonia Peruana, Iquitos, Peru; 7 Ecological Sciences, James Hutton Institute, Castle St., Aberdeen, United Kingdom; 8 Department of Biological Sciences, Florida Atlantic University, Boca Raton, FL, United States of America; 9 University Bayreuth, Soil Ecology, Bayreuth, Germany; 10 Department of Life Science and Biotechnologies, University of Ferrara, Ferrara, Italy; 11 Department of Biological Sciences, Arcata, CA, United States of America; 12 Department of Biology, Appalachian State University, Boone, NC, United States of America; 13 Zhejiang University, College of Life Sciences, Hangzhou, China; 14 Landcare Research, Hamilton, New Zealand; 15 Center for Environmental Sensing and Modeling, Singapore-MIT Alliance for Research and Technology, Singapore, Singapore; 16 Instituto de Investigaciones Forestales y Agropecuarias (CONICET-INTA), Río Negro, Argentina; 17 School of Natural and Environmental Sciences, Newcastle University, Newcastle, United Kingdom; 18 RMIT University, Applied Chemistry and Environmental Science, Melbourne, VIC, Australia; 19 Massachusetts Institute of Technology and Singapore-MIT Alliance for Research and Technology, Singapore, Singapore; 20 Department of Renewable Resources, University of Alberta, Edmonton, Alberta, Canada; 21 École Centrale de Lyon, Université de Lyon, Environmental Microbial Genomics, Laboratoire Ampère, Ecully, France; 22 Massachusetts Institute of Technology, Cambridge, MA, United States of America; 23 Department of Biology, University of Nebraska Omaha, Omaha, NE, United States of America; 24 University of Helsinki, Helsinki, Finland; 25 Natural Resources Institute Finland, Helsinki, Finland; 26 Ecosystems and Environment Research Program, Faculty of Biological and Environmental Sciences, University of Helsinki, Helsinki, Finland; 27 Institute of Landscape Ecology, Ecohydrology & Biogeochemistry Group, University of Muenster, Muenster, Germany; 28 USDA Forest Service, Northern Research Station, Grand Rapids, MI, United States of America; 29 Rochester Institute of Technology, Gosnell School of Life Sciences, Rochester, NY, United States of America; 30 Ontario Forest Research Institute, Sault Ste. Marie, ON, United States of America; 31 Department of Geography, McGill University, Montreal, Canada; 32 Laurentian University, School of the Environment and the Vale Living with Lakes Centre, Sudbury, Ontario, Canada; 33 University of Florida, Soil and Water Sciences, Gainesville, Florida; 34 Department of Microbiology, Ohio State University, Columbus, OH, United States of America; 35 Department of Biodiversity, Conservation and Attractions, Kensington, W.A., Australia; 36 Biosciences Division, Oak Ridge National Laboratory, Oak Ridge, TN, United States of America; 37 Department of Biology, California State University San Bernardino, San Bernardino, CA, United States of America; 38 Institute of Ecology and Earth Sciences, University of Tartu, Tartu, Estonia; 39 College of Science, King Saud University, Riyadh, Saudi Arabia; 40 Forest Protection Research Centre, Vietnamese Academy of Forest Sciences, Hanoi City, Vietnam; 41 USDA Forest Service, Southern Research Station, Cordesville, SC, United States of America; 42 School of Forest Sciences, University of Eastern Finland, Joensuu, Finland; 43 INSTAAR, University of Colorado, Boulder, CO, United States of America; 44 Department of Ecosystem Biology, University of South Bohemia in České Budějovice, České Budějovice, Czech Republic; 45 Department of Earth Science and Institute for the Study of Earth, Oceans and Space, University of New Hampshire, Durham, NH, United States of America; 46 Geology, Minerals, Energy, and Geophysics Science Center, USGS Menlo Park, Menlo Park, CA, United States of America; 47 State Environmental Protection Key Laboratory of Wetland Ecology and Vegetation Restoration, Institute for Peat and Mire Research, Northeast Normal University, Changchun, Jilin, China; 48 College of Forestry, Hebei Agricultural University, Baoding, Hebei, China; 49 Earth Innovation Institute, San Francisco, CA, United States of America; 50 Departments of Biological Sciences, University of Cincinnati, Cincinnati, Ohio, United States of America; 51 Department of Soil Science, College of Agriculture and Bioresources, University of Saskatchewan, Saskatoon, Saskatchewan, Canada; 52 Department of Natural Resources, Cornell University, Ithaca, NY, United States of America; 53 Nanjing University of Information Science and Technology, School of Hydrology and Water Resources, Nanjing, China; 54 Utah Valley University, Orem, UT, United States of America; University of South Florida, UNITED STATES

## Abstract

Peatlands account for 15 to 30% of the world’s soil carbon (C) stock and are important controls over global nitrogen (N) cycles. However, C and N concentrations are known to vary among peatlands contributing to the uncertainty of global C inventories, but there are few global studies that relate peatland classification to peat chemistry. We analyzed 436 peat cores sampled in 24 countries across six continents and measured C, N, and organic matter (OM) content at three depths down to 70 cm. Sites were distinguished between northern (387) and tropical (49) peatlands and assigned to one of six distinct broadly recognized peatland categories that vary primarily along a pH gradient. Peat C and N concentrations, OM content, and C:N ratios differed significantly among peatland categories, but few differences in chemistry with depth were found within each category. Across all peatlands C and N concentrations in the 10–20 cm layer, were 440 ± 85.1 g kg^-1^ and 13.9 ± 7.4 g kg^-1^, with an average C:N ratio of 30.1 ± 20.8. Among peatland categories, median C concentrations were highest in bogs, poor fens and tropical swamps (446–532 g kg^-1^) and lowest in intermediate and extremely rich fens (375–414 g kg^-1^). The C:OM ratio in peat was similar across most peatland categories, except in deeper samples from ombrotrophic tropical peat swamps that were higher than other peatlands categories. Peat N concentrations and C:N ratios varied approximately two-fold among peatland categories and N concentrations tended to be higher (and C:N lower) in intermediate fens compared with other peatland types. This study reports on a unique data set and demonstrates that differences in peat C and OM concentrations among broadly classified peatland categories are predictable, which can aid future studies that use land cover assessments to refine global peatland C and N stocks.

## 1. Introduction

Peatlands store a disproportionate amount of the global terrestrial carbon (C), covering approximately 3% of the earth’s surface yet accounting for between 15 and 30% of the world’s soil C stock [1, 2]. Around 80% of the peatlands are in temperate-cold climates in the northern hemisphere. The remaining peatlands are mostly found in tropical-subtropical climates, including south-east Asia [3–5], central Africa [6], and South America [7, 8]. Nonetheless peatlands are still often dramatically underrepresented in efforts towards comprehensive global soil databases [9–11]. This is likely due to both a lack of measurements taken to describe organic soil properties compared with mineral soils, as well as under-sampling of peatlands in remote regions.

Peatland C stocks have been most widely estimated for northern peatlands, with Gorham’s [1] estimate of 455 Gt C being the most widely accepted value. Efforts to characterize peatland C stocks have produced values that range from a low of 270 Gt C [12] to a high of 679 Gt C by Bridgham *et al*. [13] who considered all wetlands, not just peatlands. Yu *et al*. [14] reported that northern peatlands contained between 473 and 621 Gt C, tropical peatlands contained between 44 and 55 Gt C and southern peatlands contained between 13 and 18 Gt C. More recently, Nichols *et al*. [15] estimated that global peatland C stocks may exceed 1000 Gt. Regionally, Yu [16] summarized published work on northern peatlands to produce an estimate of 500 ± 100 Gt C but acknowledged that the greatest sources of uncertainty in these estimates included peat depth, bulk density and assumed peat C concentration.

Carbon stock estimates in peatlands are a function of the assumed C concentration in peat with the most widely cited mean global estimates of peat C concentration falling within a narrow range of 500–517 g kg^-1^ [1, 16], despite the fact that peat C concentrations vary widely depending upon peatland type and location and a wide range of C values have been reported both among and within individual studies [6, 17–19]. Given the large variation reported within individual studies, differences in C concentration are most likely due to differences among peatlands rather than differences in analytical methods among studies. For example, Loisel *et al*. [20] analyzed peat from 56 northern peatland sites distributed through Eurasia and North America and reported peat C concentrations of 470 ± 60 g kg^-1^, with notable regional variation in measured C concentrations. Relatively small differences in assumed “average” C concentrations can have a major impact on estimates of C stocks at the global scale. As an example, two recent meta-analysis studies have examined the potential impact of using mean global C values on two other major terrestrial C stocks (trees and dead wood) and suggested that assuming a mean C proportion of 50% may overestimate C stocks in both forests and dead wood [21, 22]. For context, Martin *et al*. [22] estimated that using a mean C concentration of 48.5% instead of 50%, reduced C stocks in CWD in tropical forest by 3 Pg C, which is similar to the entire deadwood stock in the temperate biome

An additional factor to consider is that many studies measure organic matter (OM) rather than C concentration in peat and use an assumed C:OM ratio to convert organic matter stocks into C [1]. The C:OM ratio can vary depending upon organic matter type [23], but the most widely used values typically range from 0.50–0.57 [24]. Accounting for variation in the C:OM ratio or establishing a more consistent C:OM ratio that may be linked to broadly characterized peatland categories would enable more precise estimates of C stocks when only OM data are available [23].

Peatlands also store large quantities of nitrogen (N) and can have a large impact on the global N cycle [2, 25]. Studies typically combine C:N ratios in peat with estimates of C stocks to quantify current peatland N pools [20, 26]. In a review of the literature, Yu [16] estimated that northern peatlands currently hold 10–13 Gt N based on an assumed C stock of 270 Gt C and a C:N ratio of 20–30 obtained from fen peat [27, 28]. Leifeld and Menichetti [2] estimated that N stocks in tropical peatlands amounted to 4 Gt N based on a C stock of 119.2 Gt C and a mean C:N ratio of 29.7. Other studies have shown that C:N ratios are much more variable. Based on 40 northern peatland cores sampled across North America and Eurasia including more than 3000 samples that included predominantly *Sphagnum* and non-*Sphagnum* peat, Loisel *et al*. [20] reported that C:N mass ratios varied considerably among northern peatland types and C:N ratios at individual sites were between 12 and 217 with an overall average of 55 ± 33. Lampela *et al*. [29] also found that C:N ratios in peat from a tropical peat swamp were between 27–79, which were also greater than the mean value used by Yu [16].

More recent efforts to characterize soil C and N stocks in northern regions combine empirical measurements with land-cover classification that differ in physical (bulk density and chemical) properties [30–32]. While these approaches greatly improve estimates of C and N stocks in peatlands, these studies are still based on very limited peatland chemistry data sets and are focused mostly on northern regions. In this study we work to address this gap by comparing C, N, and organic matter concentrations in peat (up to 70 cm depth) obtained from 436 cores sites ranging from acidic bogs and tropical peat swamps to more alkaline rich fens distributed across six continents and 24 countries. Peatlands were characterized into six broadly recognizable peatland classifications (ombrotrophic tropical peat swamps, minerotrophic tropical peat swamps, bogs, poor fens, intermediate fens, and extremely rich fens) that generally fall along an acidity gradient, and peat chemistry was compared across these peatland categories at depths of 10–20 cm, 30–40 cm and 60–70 cm.

## 2. Materials and methods

### 2.1. Project sampling

**Fig 1:**
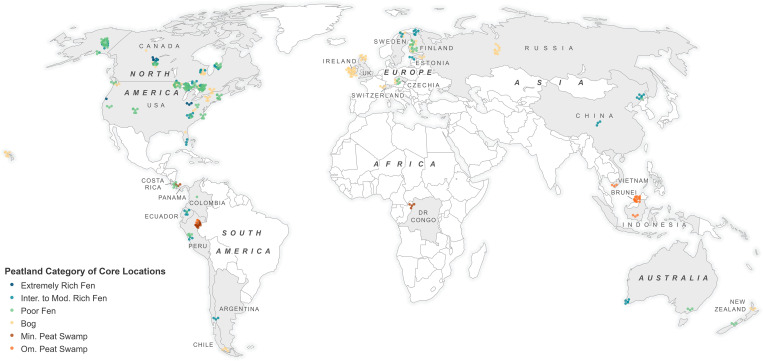
Global distribution of core sampling locations by peatland types. Points are offset to help reveal sampling intensity.

We quantified global variation in peatland C and N concentrations and organic matter content in 1226 individual peat samples at different depths, from 436 cores, collected across 24 countries and six continents (Fig 1). The samples are part of a larger project characterizing the geochemistry and microbial communities in global peatlands ([33], Global Peatland Microbiome Project). An important aim of the overall project was to generate a common data set across peatlands that was robust enough to allow global-scale comparisons but was also flexible enough to be useful for local-scale research projects at each site. Therefore, peatlands selected for sampling, specific coring locations within each wetland, and boundaries around peatlands and sampling sites within peatlands, were chosen to meet the individual needs of each collaborator using their local knowledge of hydrology, vegetation, and peat chemistry. In this study any degraded or experimental peatland sites identified by the local researcher were excluded from the analysis. The final samples included in this projected were divided into 146 collection locations, within 107 distinct peatlands. In most cases, at least three cores were sampled from a site, with cores ranging from approximately 1 m to 500 m apart within a site (<100 m in most cases), and distinct sampling sites within wetlands ranged from 10 m to 5000 m apart. The variation in distances among cores and sites primarily reflects variation in the spatial scale of habitat types among the diversity of peatlands included in the project. Peat was collected from a topographic position that would be most representative of most of the habitat at a site. Coring and collection methods included Russian peat borer, box corer, or sampled by hand using a serrated knife to achieve depth increments of 10–20, 30–40, and 60–70 cm, when possible, while cleaning sampling tools and avoiding compaction. In each case, a representative subsample of each 10 cm depth increment from a core was obtained by homogenizing the sample in a plastic bag, or by randomly pulling material from several locations across the sample, with the goal of obtaining ~50 g field moist peat per sample. Collaborators used rapid shipping methods, and when possible, shipped on dry ice or ice packs to ensure samples were minimally impacted during transportation. Samples were catalogued and stored at -20°C to prevent sample degradation prior to analysis.

### 2.2 Chemical analysis

In the lab, sub-samples were immediately oven dried at 60°C and ground to a fine powder in a ball mill. Organic matter was determined by combusting 0.1–0.5 g of dry ground peat in a 10 ml crucible at 550°C in a muffle furnace for 6 hours. Samples were then re-weighed to determine the proportion of mass loss (OM %). Peat pH was measured in a slurry at a 2:1 ratio (volume to volume) of distilled water to peat. A small number (< 20) of samples were freeze-dried by collaborators, prior to shipping to our lab; freeze dried samples were rewetted to 90% water content using distilled water and left to sit for several hours prior to measuring pH. For C and N analysis dried ground peat was weighed to within 0.0001 g on a digital balance. Approximately 75 mg of peat was placed into a formed tin foil capsule along with 150 mg of tungsten (VI) oxide powder. The tin foil was folded over sealing the sample and was compressed in a pill-shaping device. Packed samples were analyzed using an Elementar VarioMacro CNS Analyzer and precision of results was confirmed using blanks and sulfadiazine for CNS recalibration and QA standard (NIST-1515-SRM apple leaves). Laboratory processing and analyses for all samples was done consistently by the same people in Erik Lilleskov’s group at the Northern Research Station (US Department of Agriculture- Forestry Service, Houghton, MI USA) for milling and pH determination; in Nathan Basiliko’s group at Laurentian University (Sudbury, Ontario, Canada) for OM measurements and preparation for CNS analysis, and in Shaun Watmough’s group at Trent University (Peterborough, Ontario, Canada) for CNS analysis.

### 2.3 Peatland classification

To classify habitats, sites were binned into categories based on pH ranges put forth by Rydin and Jeglum [28]; bog pH <4.2, poor fen 4–5.5, intermediate and moderately rich fen pH = 5–7, extremely rich fen pH = 6.8–9. These peatland category types represent widely utilized general categories along the bog-fen gradient and range from totally precipitation fed bogs (ombrotrophic) to highly ground-water dependent (minerotrophic) fens. All distinct sampling sites were initially categorized into these four categories using the average peat pH from the 10–20 cm samples. In general, these sites could also be distinguished by considering pH and peat Ca concentration together. Final decisions on the small number of sites with pH values in overlapping regions were based on the expert opinions of the contributors who worked at those sites. Due to their ecological distinctiveness, we further separated lowland tropical peat swamps from the other sites but retained the tropical peat swamp sites within the categories provided by the pH cut-offs. This resulted in two additional habitat categories for comparison: ombrotrophic peat swamp forest (pH <4.2) and minerotrophic peat swamp forest (pH = 4.2–5.5). Peatlands are extremely diverse in character and there are a great variety of detailed classification systems (see [28]). However, it is unlikely that any one of these systems is entirely sufficient for categorizing sites that span the geographic range of our study. We believe that the simplified broad habitat categories we used provide sufficient resolution for examining the broad patterns of peat C and N chemistry that are this study’s primary interest. Although our study includes an extensive number of peatlands, from both inside and outside the biosphere’s primary peat forming regions, our data set is not necessarily directly reflective of the proportional distribution of each of these peatland types within each region. As examples of this potential bias, we note for example that we only included 10 and 11 sites from the Hudson Bay Lowlands in the Far North of Ontario, Canada and the West Siberian Lowlands respectively (Fig 1), yet these are two of the largest global peatland complexes.

### 2.4 Statistical analysis

Linear mixed models were used to examine patterns of C, N, C:N, OM, and C:OM among peatland habitats, utilizing R 3.5.2 [34]. Models for each response variable included *depth* in the peat profile, *peatland type*, and a *peatland type x depth* interaction as fixed effects. Each model also included individual peat *core*, *1 km diameter* and *100 km diameter spatial clusters* as random intercept effects, to account for samples from the same core, wetland or region being less independent from each other than samples from much more distant locations. Spatial clusters were generated with the leaderCluster package [35], using latitude and longitude coordinates for the coring locations. Our aim was to conduct large-scale comparisons among broad peatland classes, using a specific depth increment within an individual core as the fundamental sampling unit; therefore, defining individual cores, and the spatial clusters as random effects helped statistically control for local-scale variation in unmeasured factors, and controlled for differences in coring methods and sampling location selection approaches among collaborators, that are not of interest to our inference. Models were fit with the *lmerTest* package [36], and fixed effects were tested with *F*-tests utilizing the Kenward-Roger approximation. Partial *R*^*2*^ values for each fixed effect were obtained with the package *r2glmm* [37]. Post-hoc comparisons were performed in *emmeans* [38]. For each variable, each model was run with two different subsets of the data, including: 1) all cores in the data set containing data from the 10–20 and 30–40 cm depths (N = 958 samples, 479 cores), and 2) all cores in the data set with data from all three depths (10–20, 30–40, and 60–70 cm; N = 1062 samples, 354 cores).

## 3. Results

### 3.1 Peat chemistry variation with depth

**Fig 2:**
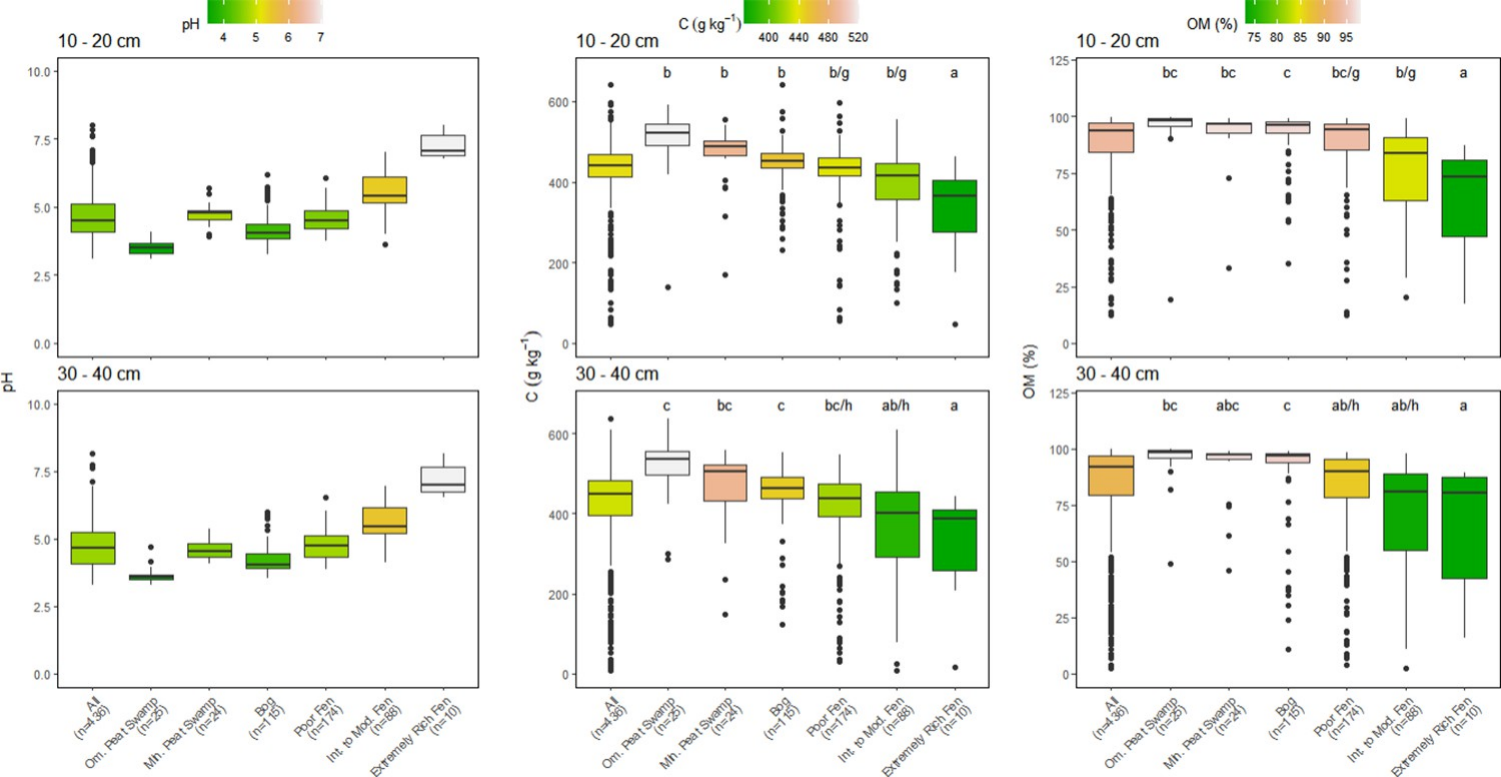
Comparison of pH, organic matter (OM) content, and carbon (C) concentrations of all sampling locations by peatland class for 10–20 cm and 30–40 cm depth increments. Box center are medians, box limits are 25^th^ and 75^th^ percentiles, whiskers are 1.5 times the interquartile range, and dots beyond whiskers are outlying points. Significant differences between peatland classes (a-e) or depth (f-h) are denoted by differing letters (p<0.05).

**Fig 3:**
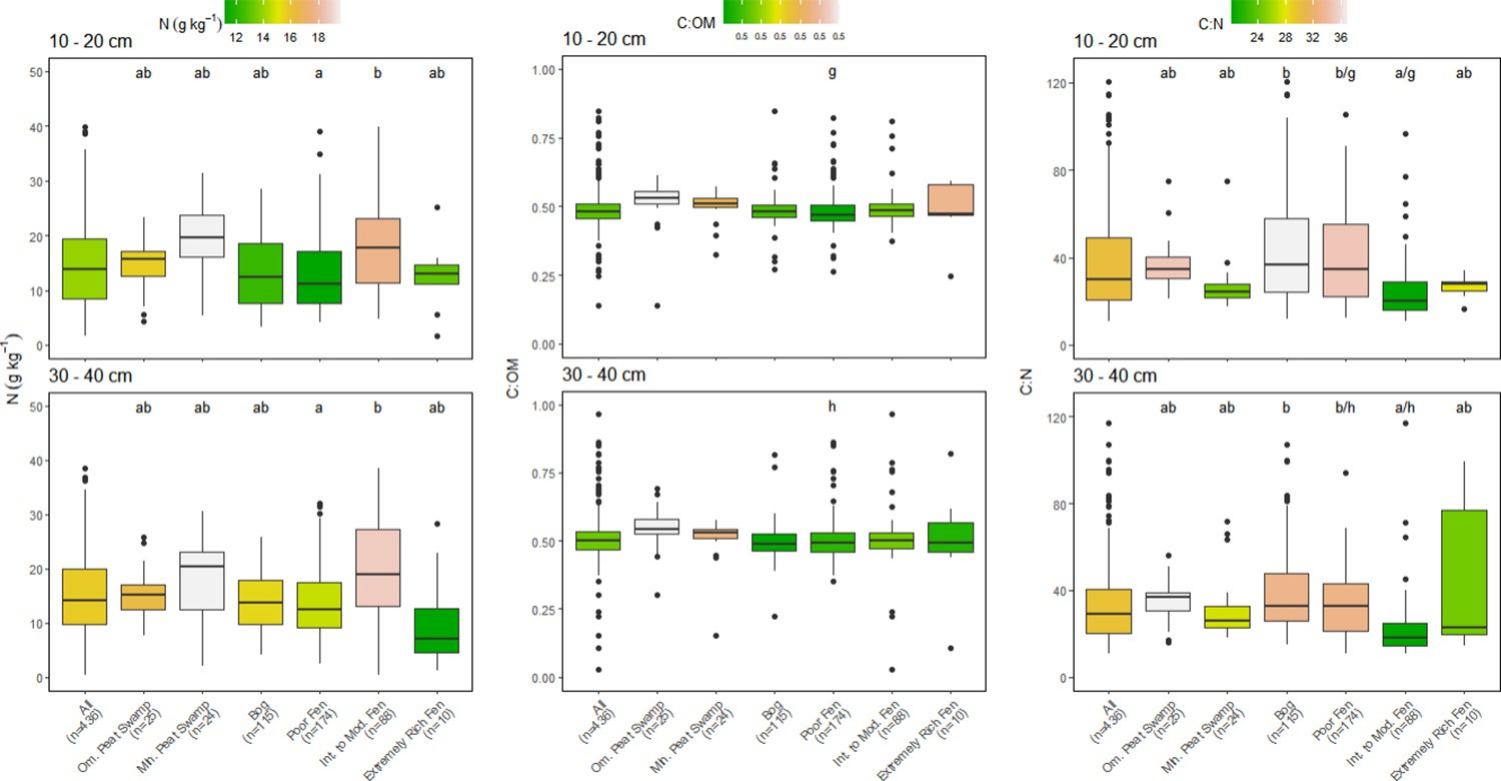
Comparison of nitrogen (N) concentration, carbon (C):organic matter (OM) ratios, and C:N ratios of all sampling locations by peatland class for 10–20 cm and 30–40 cm depth increments. Box center are medians, box limits are 25^th^ and 75^th^ percentiles, whiskers are 1.5 times the interquartile range, and dots beyond whiskers are outlying points. Significant differences between peatland classes (a-e) or depth (f-h) are denoted by differing letters (p<0.05).

**Fig 4:**
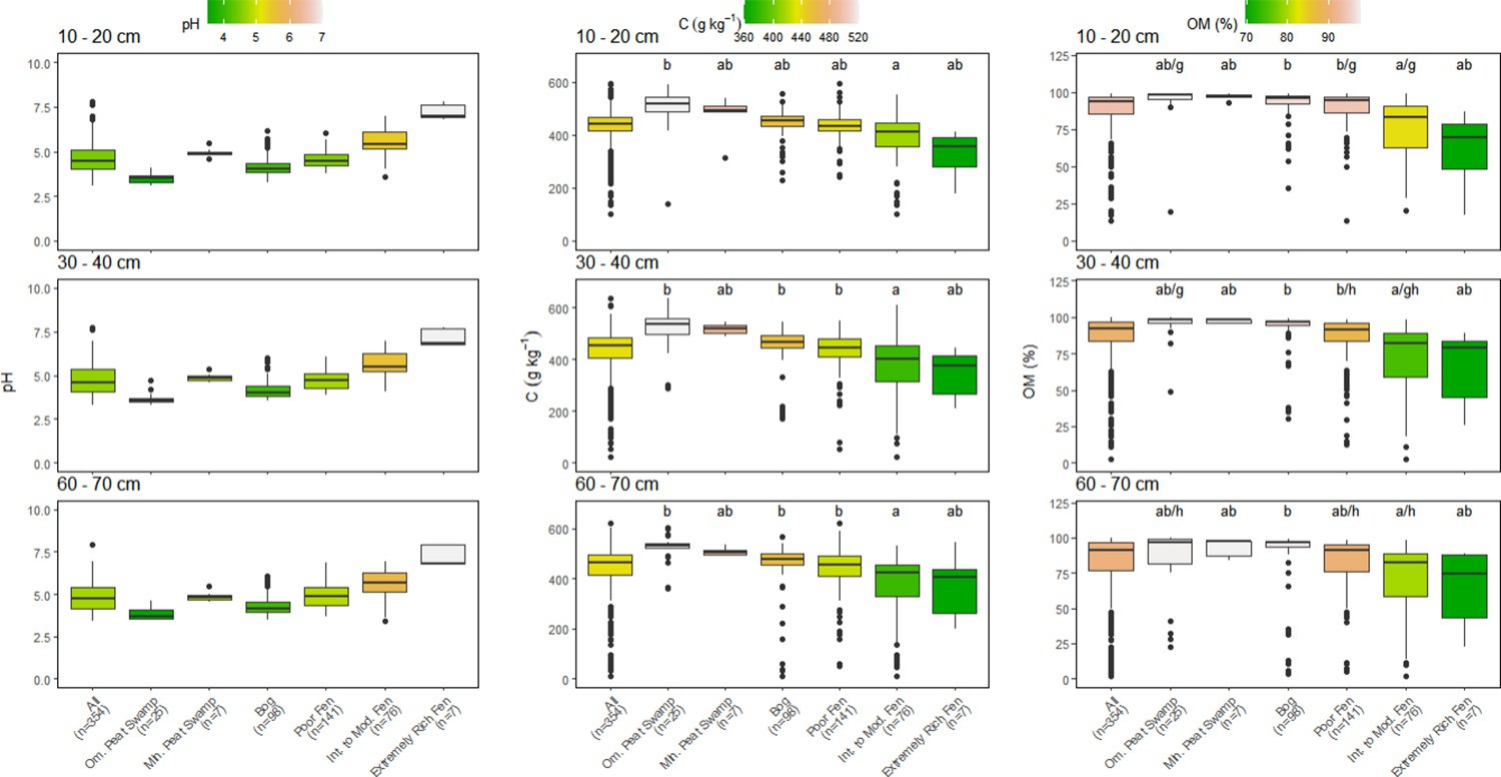
Comparison of pH, organic matter (OM) content, and carbon (C) concentrations of all sampling locations by peatland class for matching cores at 10–20 cm, 30–40 cm, and 60–70 cm depth increments. Box center are medians, box limits are 25^th^ and 75^th^ percentiles, whiskers are 1.5 times the interquartile range, and dots beyond whiskers are outlying points. Significant differences between peatland classes (a-e) or depth (f-h) are denoted by differing letters (p<0.05).

**Fig 5:**
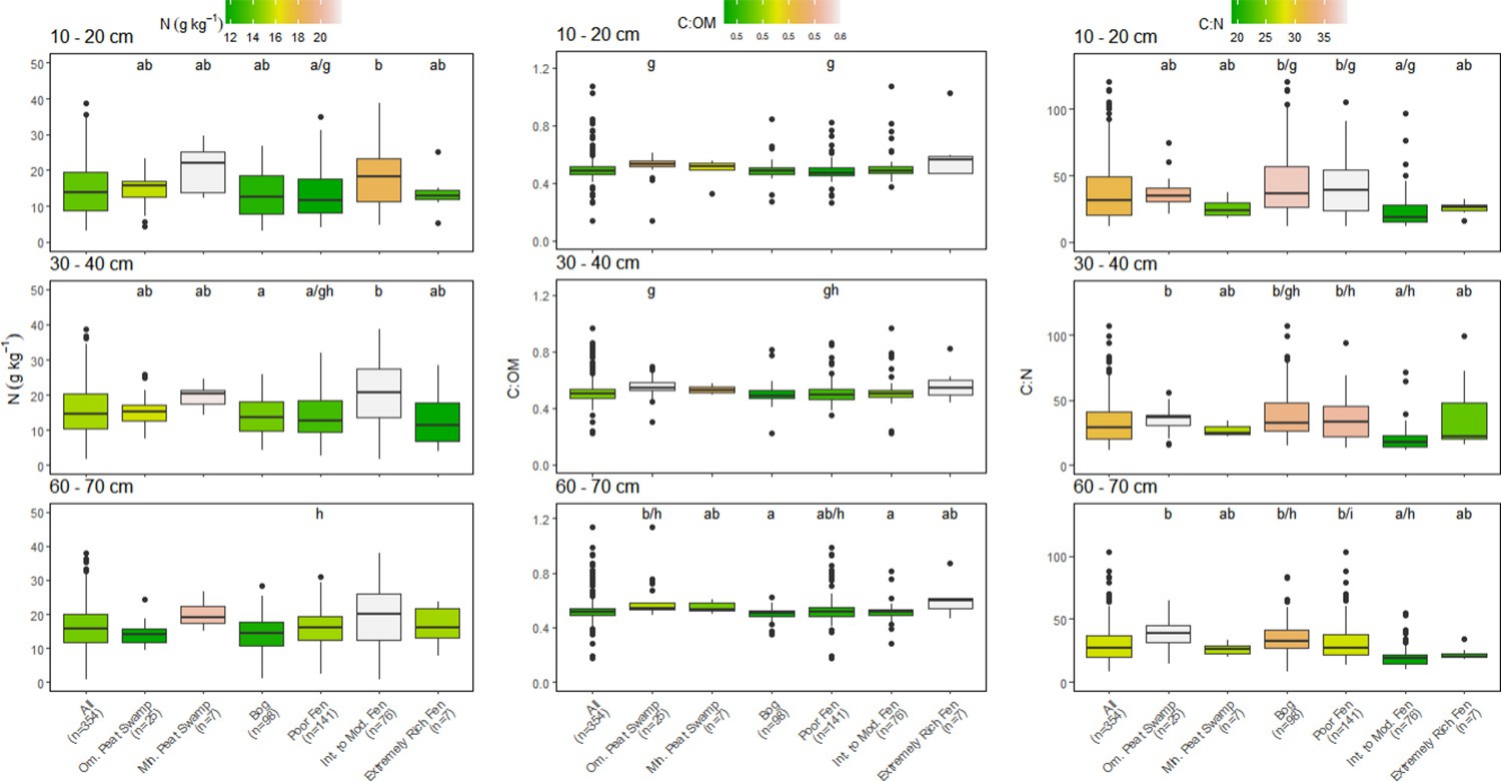
Comparison of nitrogen (N) concentration, carbon (C):organic matter (OM) ratios, and C:N ratios of all sampling locations by peatland class for matching cores at 10–20 cm, 30–40 cm, and 60–70 cm depth increments. Box center are medians, box limits are 25^th^ and 75^th^ percentiles, whiskers are 1.5 times the interquartile range, and dots beyond whiskers are outlying points. Significant differences between peatland classes (a-e) or depth (f-h) are denoted by differing letters (p<0.05).

Few significant differences among depth were found within each peatland category (Table 1). Only OM content varied significantly with depth, with values tending to decrease with depth but decreases were mostly <5% (Figs 2 and 4). The decrease in OM also resulted in small increases in the C:OM ratio with depth in some peatland categories (Figs 3 and 5). The median C concentration across all peatlands was very consistent falling between 442–464 g kg^-1^, depending on peat depth (Figs 2 and 4).

**Table 1:** Linear mixed model null-hypothesis test results for peat chemical characteristics in three versions of the dataset^a^^-^^d^.

	10-20/30-40 cm unfiltered			10-20/30-40/60-70 cm unfiltered		
	*F*	*df*	*P*	*F*	*df*	*P*
**Carbon**						
Peatland type	5.85	(5, 114.4)	<0.0001	6.12	(5, 93.1)	<0.0001
Depth	0.08	(1, 430)	0.7775	0.99	(2, 696)	0.3708
Peatland type X Depth	1.33	(5, 430)	0.2478	0.88	(10, 696)	0.5558
**Nitrogen**						
Peatland type	3.59	(5, 112.8)	0.0047	3.12	(5, 89.7)	0.012
Depth	0.16	(1, 430)	0.6929	0.93	(2, 696)	0.3969
Peatland type X Depth	0.88	(5, 430)	0.4940	0.92	(10, 696)	0.5096
**Carbon:Nitrogen**						
Peatland type	4.53	(5, 108.8)	0.0009	10.2	(5, 84.2)	<0.0001
Depth	0.04	(1, 430)	0.8469	1.95	(2, 696)	0.1434
Peatland type X Depth	3.27	(5, 430)	0.0066	1.47	(10, 696)	0.1470
**Organic matter**						
Peatland type	6.76	(5, 113.6)	<0.0001	5.94	(5, 92.9)	<0.0001
Depth	2.28	(1, 430)	0.1317	4.34	(2, 696)	0.0133
Peatland type X Depth	2.77	(5, 430)	0.0178	1.14	(10, 696)	0.3324
**Carbon: Organic matter**						
Peatland type	1.06	(5, 60.7)	0.3917	2.91	(5, 68.8)	0.0194
Depth	0.01	(1, 430)	0.9030	4.05	(2, 696)	0.0178
Peatland type X Depth	0.76	(5, 430)	0.5808	1.10	(10, 696)	0.3558

^a^ Analyses were run on two versions of the dataset: *10-20/30-40 cm unfiltered* = 10–20 and 30-40cm samples from all cores, *10-20/30-40/60-70 cm unfiltered* = 10–20, 30–40, 60-70cm samples from cores containing all three depths.

^b^All models also contained the random effects *Core*, *1km diameter spatial cluster* and *100km diameter spatial clusters*, but no hypothesis test was applied these variables. Peatland type = ombrotrophic peat swamp, minerotrophic peat swamp, bog, poor fen, intermediate to moderate rich fen, extremely rich fen.

^c^Carbon:Nitrogen and Carbon:Organic matter were log10 transformed prior to analyses.

^d^Denominator degrees of freedom were estimated with the Kenward-Rogers approximation, using Type III sums of squares.

### 3.2 Peat chemistry variation among peatland categories

Much larger differences in peat chemistry were evident among the six broad peatland categories than were found with depth within each category (Figs 2–5). Within each peatland category, C, N and OM values measured in the 10–20 cm and 30–40 cm depths were almost identical using the entire data set or the abbreviated data set that contained all three depths but had fewer sites (Figs 2–5). The six peatland categories reflect an increasing pH gradient: ombrotrophic tropical peat swamps <bogs<poor fens<minerotrophic tropical peat swamps<intermediate fens<extremely rich fens (Figs 2 and 4). There was considerable variation in median C concentrations among peatland categories (Figs 2 and 4). Highest median peat C concentrations were found in ombrotrophic and minerotrophic tropical peat swamps, followed by bogs and poor fens, with considerably lower median C concentrations recorded in intermediate and extremely rich fens (Figs 2 and 4).

Patterns in OM content across the six peatland categories were very similar to differences observed in peat C concentration (Figs 2 and 4). Median OM values across all peatlands were between 91.3–93.7% depending on peat depth (Figs 2 and 4). As with C concentration, there was considerable variation among peatland categories and OM values followed a similar pattern to C among the peatlands. Peat OM values were highest in tropical peat swamps, bogs, and poor fens, but were much lower and more variable with depth in intermediate and rich fens (Figs 2 and 4). The similar pattern in peat C concentration and OM values across peatlands resulted in generally consistent C:OM ratios across the six peatland categories, although the C:OM ratio in ombrotrophic tropical peat swamps was higher than the other peatland categories at all depths (Figs 3 and 5). Peat C:OM ratios also tended to be slightly higher in the deepest peat samples that we assessed (Figs 3 and 5). Averaged across all sites the median C:OM ratio in peat was between 0.48–0.51, depending on peat depth.

Peat N concentrations and C:N ratios also differed significantly among the six peatland categories, but patterns were less consistent than were found for C and OM (Figs 3 and 5). Averaged across all sites, median N concentrations were 13.9–15.6 g kg^-1^ and median C:N ratios were 27.2 and 31.6 depending on depth, with slightly higher N concentrations and lower C:N ratios measured in deeper peat (Figs 3 and 5). Minerotrophic tropical peat swamps and intermediate fens generally had higher N concentrations than the other peatland categories (approximately 20 g kg^-1^ versus 15 g kg^-1^). Peat C:N ratios were generally lowest in minerotrophic peat swamps and both intermediate and extremely rich fens except at the 30–40 cm depth (Figs 3 and 5).

## 4. Discussion

In our global data set, peat C concentration exhibited little variation with depth (to 70 cm) but varied considerably among the six peatland categories that we used in this study, with tropical peat swamps, bogs and poor fens having higher C concentrations than intermediate and extremely rich fens. Linking differences in peat chemistry to peat or land cover classification is essential in upscaling studies designed to characterize regional or global C stocks [32]. Loisel *et al*. [20] identified five peatland categories (*Sphagnum*, herbaceous, woody, humified and brown moss) in their synthesis of northern peatlands and found that mean C concentrations for each category varied between 460–509 g kg^-1^ but did not clearly sample or distinguish minerotrophic fens that have the lowest C concentration in our data set. The difference in the types of peatlands used in our study compared with Loisel *et al*. [20] is likely the major reason for the differences in peat C concentration, highlighting the need for additional sampling efforts to better constrain global peatland C stocks.

In our study, all peatlands were placed into one of six broad categories (bogs, poor fens, moderate to intermediate fens, extremely rich fens, ombrotrophic tropical peat swamps and minerotrophic peat swamps) that encompassed a wide variety of peatland types. Differences in peatland classification are also widely evident in the literature (e.g., [20]) and can be heavily debated, but in our broad classification, peatlands categories are generally distinguished by differences in pH easily allowing other peatland classifications to be placed into context with this study. Chimner *et al*. [17] similarly reported that C concentrations vary among peatland types, reporting that C concentrations in upper peat were between 457 g kg^-1^ in *Sphagnum* dominated peat with a pH of 3.9 to 387 g kg^-1^ in peatlands dominated by ash (*Fraxinus*) with a pH of 5.8. We acknowledge that some peatland types may not fall along the broad acidity gradient that we identify here, and we could have attempted to divide our four categories along the bog-fen gradient into treed and non-treed sites. Nevertheless, the pattern we observed in our classification suggests that categorizing peatlands simply on their degree of ombrotrophy (bog–rich fen gradient) is very useful for quantifying C and N in peatlands, especially when combined with improved land cover classification [31, 32].

The predominant reason for the difference in C concentration among peatland types is due to varying amounts of ash (inorganic material) in peat as patterns in OM values are very similar to patterns observed for C concentration. Intermediate and extremely rich fens contain a much greater mineral fraction than bogs or peat swamps, which is reflected in the much lower OM values, especially in the deeper samples. As a result, the median C:OM ratio was quite similar among peatland categories at the upper depths, with a median value in the global data set of 0.48 to 0.51 (depending on depth), which is within the range reported in other studies [1, 24], but differs somewhat from values reported by Kasozi [23] who established C:OM relationships for carbonatic and organic soils from Florida and Puerto Rico and Spodosols from Florida, which contained generally higher ratios. We also noted that ombrotrophic tropical peat swamps tended to have higher C:OM ratios that other peatland categories, especially at the 60–70 cm depth where the median C:OM ratio was >0.7. This was not the case for minerotrophic tropical peat swamps in our data set. Previous studies have shown that *Sphagnum*-derived peat that is more typical in acidic northern peatlands has a lower C:OM ratio than peat derived primarily from herbaceous vegetation and woody plants [20, 39]. Indeed, Verbeke [33] used FTIR to show that concentrations of carbohydrates decreased relative to aromatics in samples taken from the same sites as those used in our study. This would result in a loss of organic O and C and a relative increase in C-C bonding resulting in more C relative to OM in deeper peat. In contrast, the high C:OM ratio found at deeper depths in intermediate fens is likely due to the high mineral content and such large deviations from the typical C:OM ratio has previously been noted in samples with a high ash content [23]. In this case the additional C measured by combustion is most likely from carbonate rich minerals that are present in the more alkaline fens [40].

Patterns in median N concentrations and C:N ratios in peat also varied significantly among peatland type. While our median C:N ratios in peat across all sites were between 27.2–31.6 (depending on depth), median peatland categorical values ranged from lows of around 20–25 in base rich peatlands to 30–40 in more acidic bogs, fens, and tropical peat swamps. Wang *et al*. [41] similarly reported a wide variation in near surface (upper 50 cm) peat C:N ratios, with bogs having a C:N ratio of 42.0 ± 1.3, compared with 28.8 ± 0.6 in rich fens. It has been shown that if the C:N ratio of organic matter is <20 then mineralization will generally take place and at values >30 then immobilization will occur although this can vary based on site characteristics and litter type or initial litter quality [42]. A high C:N ratio in bogs, poor fens and ombrotrophic tropical peat swamps therefore reflects a high rate of C sequestration stressing the importance of preserving natural peatlands to sequester C as opposed to attempting to build up C stocks in mineral soils that have lower C:N ratios and would require N fertilization [2]. If the lower C:N ratio in the base rich peatlands is associated with higher rates of decomposition and loss of organic matter it may additionally contribute to the generally lower C and OM values in these peatland categories.

Variations in peat C and N concentration (and C:N ratios) among these peatland types are due to several factors including differences in vegetation composition (litter inputs), litter decomposition rates [43], acidity [44] and peat mineral content. Mean N concentrations in vegetation are generally similar in bogs and fens, with the notable exception of *Sphagnum* that has much lower N concentrations and higher C:N ratios than most other peatland vegetation [43, 45]. Because *Sphagnum* is the dominant vegetation in bogs and poor fens compared with richer fens and peat swamps it plays a major role in the low N concentration and high C:N ratio in peat in these systems [20]. In addition, in all peatlands, N is resorbed from foliage during senescence; hence C:N ratios in fresh litter are considerably higher than in live foliage [43, 46]. During decomposition, C is lost through microbial action and the C:N ratio of peat decreases [41, 47]. In the more acidic bogs and poor fens in northern peatlands, decomposition rates are much slower and peat C:N ratios in upper peat are consequently higher than values found in less acidic intermediate and rich fens where decomposition is more pronounced [44, 46, 48].

Most previous estimates of global C and N stocks are estimated using data from northern peatlands as they account for 80% of global peatlands and have been historically more widely studied [1]. The presence of large peatland C stocks in low-latitude regions has been reported to be due to differences in peat C chemistry compared with northern peatlands. As with northern peatlands, there can be a wide variation in tropical peat characteristics [49], but in general, near-surface low-latitude peat has lower carbohydrate and greater aromatic content than near-surface high-latitude peat, with a greater resistance to decomposition [50, 51]. Such differences may additionally explain the higher C:OM ratio found in our ombrotrophic tropical peat swamp habitat category.

Even within northern peatlands, considerable regional variation in mean C and N concentrations in peat exists. Loisel *et al*. [20] for example, reported that mean peat C concentration varied from a low of 360 g kg^-1^ in eastern Russia and Asia to a high of 540 g kg^-1^ in the eastern European Islands. Similarly, mean N concentrations in peat are between 7.0 g kg^-1^ in continental Europe and 16.0 g kg^-1^ in several regions including Hudson Bay and James Bay, western European islands and Western Russia [20].While we do not rule out some regional variations in peat chemistry within our peatland categories and our data set is not designed to test regional patterns, it appears that they are likely less than differences found among our six broad peatland categories and would mean than peat C and N concentrations reported in near surface peat here are applicable to peatlands worldwide if they can be appropriately categorized. These efforts would be aided by the acquisition of additional data, especially in under-sampled regions and the continued development of land cover classification using remote sensing techniques and modelling efforts [52, 53] to better characterize both peatland area and type.

## 5. Conclusions

We established six broad peatland categories, consisting of ombrotrophic tropical peat swamps, minerotrophic tropical peat swamps, bogs, poor fens, intermediate fens, and extremely rich fens, that fell along an increasing pH gradient and were sampled in 24 countries across six continents. Peat C, N and OM values were relatively consistent within each category but varied considerably among the six peatland categories. Peat C concentrations were highest in bogs, poor fens and tropical peat swamps, while N concentrations were highest in minerotrophic peat swamps and intermediate fens. The C:OM ratio exhibited less variation among peatland category suggesting that differences in mineral content were largely responsible for differences in C concentration among peatlands. Peat C and C:N ratios in our data set followed a predictable pattern among broadly classified peatland categories, and data sets such as these may be used to better constrain global assessments of peat C and N in surface peat, especially when coupled with improved land cover classification.
